# Identifying the Subtypes and Characteristics of Mental Workload Among Chinese Physicians in Outpatient Practice: A Latent Profile Analysis

**DOI:** 10.3389/fpubh.2021.779262

**Published:** 2021-11-24

**Authors:** Dehe Li, Yinhuan Hu, Hao Chen, Ximin Zhu, Xiaoyue Wu, Jiayi Li, Zemiao Zhang, Sha Liu

**Affiliations:** School of Medicine and Health Management, Tongji Medical College, Huazhong University of Science and Technology, Wuhan, China

**Keywords:** COVID-19, latent profile analysis, mental workload, mental health, physicians, outpatient care, communication, China

## Abstract

**Objective:** The purpose of this study is to investigate the mental workload level of physicians in outpatient practice since the normalization of prevention and control of the COVID-19 pandemic in China and explore the subtypes of physicians regarding their mental workload.

**Methods:** A cross-sectional survey of 1,934 physicians primarily in 24 hospitals in 6 provinces in Eastern, Central, and Western China was conducted from November 2020 to February 2021. A latent profile analysis was performed to identify clusters based on the six subscales of the Chinese version of physician mental workload scale developed by our research team. Chi-square tests were performed to explore the differences in demographic characteristics of the subtypes among the subgroups, and multinomial logistic regression analysis was further conducted to identify the determinants of the subtypes of physicians.

**Results:** Overall, the participating physicians reported high levels of task load but with high self-assessed performance (68.01 ± 14.25) while performing communication work tasks characterized by direct patient interaction in outpatient clinics. About 33.8% of the participating physicians were identified as “high workload and high self-assessment” subtype, compared to 49.7% “medium workload and medium self-assessment” subtype and 16.4% “low workload and low self-assessment” subtype. Physicians in “high workload and high self-assessment” subtype had the highest mean mental workload score. Physicians who were female, younger, married, worse health status, those who had lower educational level and an average monthly income of 5,001–10,000 RMB, those who worked in tertiary A hospitals, more hours per week and more than 40 h per week in outpatient clinics, and those who saw more outpatients per day, and spent more time per patient but with higher outpatient satisfaction were more likely to belong to “high workload and high self-assessment” subtype.

**Conclusion:** Our findings can help provide a solid foundation for developing targeted interventions for individual differences across physicians regarding their mental workload. We suggest the hospital managers should pay more attention to those physicians with characteristics of the “high workload and high self-assessment” subtype and strengthen the management of the workload of this subtype of physicians to reduce the risks of their mental health, and to maintain their high work performance in outpatient clinics.

## Introduction

### Workload

Internationally, there has been a focus on the topic of the relationship between physicians' workload and their health (1), and their physical and mental health is closely related to their workload (1, 2), especially during the current ongoing COVID-19 (3). Excessive workload not only impacts physicians' health (2, 4), but also contributes to an inferior quality of care service (5) and thereby affects patient satisfaction and safety (6, 7). During the current COVID-19 pandemic, the high workload of healthcare workers on the frontline is a major concern for efficient health care, patient safety, and fatigue, burnout, the physical and mental health of healthcare workers (8, 9). Compared to SARS-CoV in 2003 and MERS-CoV in 2012, a higher infectivity and transmissibility of SARS-CoV-2 might further increase the workload burden for healthcare workers on the frontline, thereby threating their mental health even more (10).

Workload is generally thought to be a multidimensional and multifaceted construct, as the ratio of demands to available resources (11). Apart from the objective workload, one aspect of workload includes the subjective psychological experiences of the human operator while performing a task under a specific environmental or operational condition, namely mental workload (11). The assessments and management of mental workload was recommended by the European Pact for Mental Health and Welfare to promote physical and mental well-being (12). The National Aeronautics and Space Administration Task Load Index (NASA-TLX) provides the most widely accepted and validated theoretical framework (including mental demand, physical demand, temporal demand, effort, own performance, and frustration level) to measure subjective workload that an individual perceives (13), and is also used to quantify perceived workload of healthcare workers (2). Given that there was an urgent demand for managing the variety of human factors that influence the mental health of healthcare workers and that thus compromise pandemic control (14), most current studies have predominantly addressed the assessments of the mental workload of frontline health workers, especially for frontline nurses aiding the COVID-19 pandemic, especially in pandemic regions, in turn, to provide targeted guidance for developing interventions for the government and hospital mangers to facilitate the mental health of frontline healthcare workers and the quality of care in the COVID-19 pandemic, however, the mental workload level and its associated with factors among frontline physicians likewise aiding in the COVID-19 pandemic were rarely reported separately (6, 15, 16). A high mental workload is a psychological stress factor taking up part of an individual's naturally limited working memory, and ultimately leads to fewer cognitive resources being available. Hence, accurate assessment of physician mental workload is also of great importance to manage stressors, and thereby improve work performance.

### Background

As the national epidemic prevention and control battle against the COVID-19 pandemic has achieved phased success, the China's epidemic prevention and control entered a “normalization” stage since April 29, 2020 (17), and subsequently, Chinese physicians including frontline physicians aiding in the COVID-19 pandemic have gradually returned to the normal role of the delivery of medical services for patients in outpatient clinics. According to the new data from National Health Commission of the People's Republic of China, the total number of annual outpatient visits nationwide in China in 2020 has decreased 11.2% than that in 2019, whereas the medical service requirements of patients suppressed by the COVID-19 pandemic lasted for at least 5 months (18), demonstrating that a great number of accumulated medical demands of patients might have been releasing in traditional hospitals after the rest of the time in 2020, although domestic internet hospitals have been devoted to meeting the demands of patients for light inquiry services during the pandemic; and hence, physicians may have to undertake heavier outpatient workloads than over the same period before the pandemic, since the normalization of prevention and control of the COVID-19 pandemic in China. In addition, there being still a trend of Chinese patients tending to go to high-level hospitals for high-quality medical services even for mild symptoms to date, and a trend of the increasing utilization of medical services by patients (19), might further contribute to increased outpatient workload burden for these physicians in high-level hospitals. Heavy outpatient workload can not only affect physicians' health, resulting in burnout, fatigue or anxiety, but also further lead to an inferior quality of medical services to outpatients, ultimately affecting patient satisfaction as well as patient safety. Also, opportunely assessing physician workload is an important issue in managing their workload and stressors, as well as further developing targeted interventions to support them. Hence, this study focused on the assessment of physician workload in outpatient clinics.

Previous studies often simply adopted several objective workload indicators (for example, the number of patient visits, daily visits per physician, weekly work hours) for assessments of Chinese physicians' workload (20–22), while ignoring physicians' mental workload, an important part of physicians' workload. Mental workload has been regarded as one of the most critical occupational risk factors resulting in burnout or anxiety (11). However, there has been little research on the mental workload level and its characteristics among physicians in outpatient clinics to date, especially in China, though several studies addressed the development of mental workload scale for Chinese physicians (2, 23). Mental workload is also used as a more comprehensive indicator than the simple quantity of work tasks for predicting the mental health and work performance of frontline healthcare workers aiding in the current COVID-19 epidemic (8). If we also wish to reduce the risk factors affecting these physicians' mental health and to improve their work performance in outpatient clinics, we need a more accurate understanding of their mental workload. When drawing insights from previous studies that have provided a solid basis for the present study, we should also seek to go a step further to identify the different subtypes of mental workload among these physicians, and determine the characteristics and determinants of the different subtypes to, in turn, to develop targeted interventions for individual differences across physicians to facilitate their mental health and improve their work performance in outpatient practice. Hence, whether there exist different mental workload clusters in physicians and how to identify these clusters are rather worthwhile to explore. However, little is also known about the subtypes and its characteristics of mental workload among physicians, especially in outpatient practice in previous studies. According to the human-based archetype of mental workload proposed by Jafari et al., resource supply, task demand and individual characteristics were the key factors influencing mental workload (24). Therefore, related individual characteristics related to the mental workload of physicians might be vital for identifying the characteristics of the subtypes.

### Study Aims

The main objective of this study is to investigate the level of mental workload among physicians in outpatient clinics in China, and to identify the subtypes of mental workload among these physicians through latent profile analyses, a rather novel method in the mental workload research among physicians. The specific objective is to identify the characteristics of subtypes and determine the factors associated with these subtypes mainly based on the demographic variables the demographic questionnaire. Our current understanding about the subtypes of mental workload among physicians is quite limited. Previous studies often adopted a variable-centered analytical approach for assessments of physicians' mental workload (25, 26). Such kind of study, although important, has failed to reveal the different facets of mental workload among physicians. This study fills the gap in the literature by employing latent profile analyses, which can help increase the understanding of the mental workload of Chinese physicians in outpatient clinics, and further identify the different subtypes of physicians who would otherwise be missed in single indicators.

## Methods

### Study Design

#### Study Sampling

This study used stratified convenience sampling to select physicians in Eastern, Central, and Western China. To ensure sufficient representativeness, two provinces were selected in the Eastern, Central, and Western regions at the time of sampling, respectively, that is, a total of six provinces were selected. Specifically, Guangdong and Zhejiang provinces were selected in Eastern China, and Hubei and Henan provinces were selected in Central China. In Western China, Chongqing municipality and Guangxi Zhuang autonomous region were selected. In the selected provinces, typical sampling was then applied to select two tertiary public hospitals and two secondary public hospitals in each province. That is, a total of 24 public hospitals were mainly selected nationwide in China, including 12 tertiary and 12 secondary public hospitals. Among the selected hospitals, internal, surgical, obstetrics and gynecology, and pediatrics were further selected as main research departments, where targeted physicians were selected by random sampling.

#### Study Population

Given that the main purpose of this survey study was to investigate the mental workload level of physicians in outpatient practice, the setting of the research was confined to the consulting room in outpatient clinics, and therein all participating physicians who just provided medical services to outpatients in outpatient clinics after the COVID-19 pandemic were included in this survey. Moreover, the target population included physicians who had to have been working for at least 4 months in the outpatient clinics, and those who had to be employed full-time for at least 1 year in their current medical institution; whereas physicians who provided medical services to outpatients in outpatient clinics for <4 months, those who only provided inpatient service, and those who were graduate students or trainees were excluded in this study.

In the consulting room, the work tasks physicians performed, associated with their workload mainly included “communication work tasks” and “non-communication work tasks” (27); and compared to non-communication work tasks characterized by paperwork, communication work tasks characterized by direct patient interaction that mainly require physicians' brain resources, might be more highly correlated with their mental workload. Given that different types of workload resulting from different demands (“task load”) and resources (28), this study mainly investigated the workload physicians perceived while performing communication work tasks in outpatient clinics, to reflect their actual or perceived workload in outpatient practice. In our prior study, these communication work tasks mainly consisted of inquiry of medical history, explanation of medical examinations, explanation of outpatient treatment, explanation of health conditions, health guidance, and provision of information about procedures of admission/outpatient operation (27).

### Measurement Tool of Mental Workload

Mental workload data were obtained using the Chinese version of physician mental workload scale developed by our research team in 2018 based on the combination of dimensions of the NASA-TLX and Subjective Workload Assessment Technology (SWAT) frameworks (2). The Chinese version of physician mental workload scale has been verified to have good reliability and validity, indicating a reliable tool for measuring or diagnosing the mental workload of Chinese physicians that comprises six dimensions and 12 items regarding different aspects of workload (mental demands, physical demands, temporal demands, perceived risk, frustration level, and performance) (2). In this scale, all items are rated on a 10-point bipolar scale that ranges from 0 to 100. For five of the six dimensions, i.e., mental demands, physical demands, temporal demands, perceived risk, and frustration level, a score of 0 indicates the lowest task load, whereas the dimension of performance is reverse-scored, with a score of 0 indicating the most successful performance of the task and the highest level of satisfaction with his/her performance, and the lowest task load. In this study, the average score of all items of a corresponding dimension was the dimension score, whereas each dimension score was multiplied by the weight of the corresponding dimension (that is, weight dimension score), and the sum of the scores was the total score of mental workload.

Specifically, the measurement tool included three parts in this study. The first part included 6 dimensions and 12 items of the above physician mental workload scale; these dimensions were further compared two by two making the participants able to select the dimension with the most impact on their workload to collect the weights of each dimension in the second part, and therein the weight of each dimension was equal to the number of times that dimension was selected divided by 15. The third part was designed to collect the demographic information of the participants, including gender, age, marital status, educational level, average monthly income, professional title, working years, working years in the current medical institution, area, hospital level, hospital nature, personnel, department, working hours per week, outpatient working hours per week, number of outpatients serviced per day, amount of time spent per patient, self-rated health status, and self-rated outpatient satisfaction. Self-rated health status was measured by the question on a scale of 1 (very poor) through 5 (very good) that “how do you rate your overall health status?” Self-rated outpatient satisfaction was measured based on the question on a 20-point bipolar scale that ranges from 0 to 100 with 0 indicating the lowest outpatient satisfaction, that is, “how many scores you perceive that your outpatients rate for your outpatient services?” and we further divided those with a score of 30 and below into the low satisfaction group, 40–60 into the medium satisfaction group, and 70 or more into the high satisfaction group based on the 0-to-10 numeric pain rating scale (29).

Then, we performed a pilot survey on site in October 2020, to validate the measurement tool in 10 physicians who just finished the provision of the outpatient services in the outpatient clinic of a tertiary public hospital in Wuhan, Hubei. They made comments on the scale, the clarity and level of detail of the outpatient work tasks, and the general design of the survey. Subsequently, context-specific adjustments were made to improve the accuracy and clarity of the questionnaire according to the feedback from the pilot survey. Due to the impact of the COVID-19 pandemic in 2020, we further used the web-based survey tool called wenjuanxing, to create an electronic questionnaire with which to survey physicians.

### Data Collection

This survey was carried out from November, 2020 to February, 2021. To improve the efficiency of data collection in the selected hospitals, a unique two-dimensional code of the electronic questionnaire was generated for each hospital. Prior to the beginning of the survey, an informed consent of the outpatient managers in each selected hospital was requested and obtained, and they were invited and volunteered to play the role of the project manager in their hospitals in this survey. To recruit physicians in main research departments selected in this study, we then sent the unique two-dimensional code of the electronic questionnaire to these outpatient managers of the corresponding hospital, and subsequently, they sent the two-dimensional code to the targeted department groups of physicians via WeChat or Tencent QQ group, and physicians who met the inclusion criteria for the targeted population were further invited to participate in this survey. Participants could scan the two-dimensional code of the electronic questionnaire using their phones to access and complete the electronic questionnaire. Before the survey, we introduced the purpose of the survey, provided the definition of mental workload and its outpatient work tasks involved, and guaranteed that the survey data would not be used for other purposes. After an individual's consent was obtained, the survey was conducted accordingly. A WeChat or Tencent QQ account and mobile Internet Protocol address could be used to complete the questionnaire only once. Moreover, to improve the scale of the targeted physicians, these physicians who completed the questionnaire were also encouraged to share the survey website link to their Wechat Circle of Friends, WeChat or Tencent QQ group, where some physicians who met the inclusion criteria for the targeted population could participate in this survey. The study was approved by the Ethics Committee of Tongji Medical College of Huazhong University of Science and Technology (No. IORG0003571).

### Statistical Analysis

Descriptive statistics were used to summarize data on demographic characteristics, mental workload and its dimensions of the participating physicians. Data were summarized as frequencies (n) and percentages (%) for categorical variables.

To identify the subtypes of mental workload among physicians, exploratory latent profile analysis (LPA) was performed using the six dimensions of mental workload in this study. LPA, a person-centered statistical approach, provides a method to group individuals with similar patterns of personal and professional characteristics, traits or behaviors into non-overlapping profiles based on their responses to a set of continuous observed indicators (30). Previous studies found that LPA was used as a reliable and feasible approach to the identification of different facets of mental workload among individuals (31), including frontline nurses aiding in the COVID-19 pandemic (6). Therefore, LPA can be used to identify the patterns of mental workload among Chinese physicians in outpatient clinics likewise. Data for the six dimension indicators were input into the LPA, with one class initially and additional classes added incrementally, until a unique solution could not be determined with the maximum likelihood method.

We tested different models that categorized the subtypes of mental workload into one, two, three, four, five, six and seven Classes. The best fit model was mainly identified using the following model indexes: Akaike information criterion (AIC), Bayesian information criterion (BIC), sample-size Adjusted BIC (ABIC), Lo-Mendell-Rubin (LMR), adjusted likelihood ratio test and bootstrap likelihood ratio test (BLRT) and Entropy (32). A lower value of AIC, BIC and ABIC indicates better fitness of data into the estimated model (32). LMR and BLRT compare the model fit between two neighboring models (for example, k-1class model vs. k-class model), and a lower *P*-value represents that the k-class model fits the data better than the k-1-class model (32). Entropy assesses the accuracy of classification, with values closer to 1 indicating better classification (32). To avoid over-stratification, the smallest group should have a minimum of 5% of the total sample (32). A three-class model was identified in the LPA. Each participating physician was assigned into one of the classes of mental workload of physicians with the highest probability.

Chi-square (χ^2^) tests were then used to explore the differences in the three classes across demographic characteristics, and the differences in mental workload and its dimensions among different Classes were tested using one-way analysis of variance (ANOVA) tests. Multinomial logistic regression analysis was applied to identify the significant factors predicting the three subtypes of physicians regarding their mental workload, and therein the demographic variables were set as independent variables. Data analyses were performed using Mplus software (version 7.0) and STATA 15.0 software. *P* < 0.05 was considered statistically significant.

## Results

### Participant Characteristics

In total, 2,038 responses were received; of these, 104 responses were excluded because the time taken to answer the questionnaire was <60 s, or because they were not physicians, or they were physicians, but did not provide medical services to outpatients in outpatient clinics. Of the 1,934 participating physicians, 45.9% were female, the average age of them was 38.12 years (SD = 8.38 years, range: 20–77 years), 82.0% were currently married, and 74.9% were from tertiary hospitals. Furthermore, among these participating physicians, 51.9% had a postgraduate degree or higher, 38.0% were from Eastern China, 54.9% worked 41–60 h per week and 35.8% worked more than 60 h per week; the average time per week working in outpatient practice was 19.50 h (SD = 14.18 h, range: 3.5–60 h) and the physician saw an average of 43.20 (SD = 24.81, range: 10–160) patients per day in outpatient clinics. In addition, almost half (46.6%) of the participating physicians rated health status as moderate, and 87.9% of them rated outpatient satisfaction as high. Detailed demographic characteristics of the 1,934 participating physicians are presented in Table 1. Moreover, the total mean score of workload the participating physicians perceived ranged from 17.11 to 100.00 while performing communication work tasks characterized by direct patient interaction, and the mean score was 68.01 (SD = 14.25) and therein the highest weighted score in six dimensions was mental demands, followed by the perceived risk, temporal demands, frustration level, physical demands, and performance (seen from Table 2).

**Table 1:** Demographic characteristics of the participating physicians (*N* = 1,934).

**Characteristics**	**Number (%)**	**Characteristics**	**Number (%)**
Gender		Hospital level	
Male	1,047 (54.1)	Tertiary A hospital	1,234 (63.8)
Female	887 (45.9)	Tertiary B hospital	215 (11.1)
Age (years)		Secondary hospital	447 (23.1)
20–30	433 (22.4)	First-tier hospital	38 (2.0)
31–40	852 (44.1)	Department	
41–55	587 (30.4)	Internal	585 (30.2)
>55	62 (3.2)	Surgical	481 (24.9)
Marital status		Obstetrics and gynecology	192 (9.9)
Unmarried	305 (15.8)	Pediatrics	163 (8.4)
Married	1,585 (82.0)	Other	513 (26.5)
Divorced	36 (1.9)	Working hours per week	
Widowed	8 (0.4)	≤ 40	180 (9.3)
Educational level		41–60	1,062 (54.9)
PhD	228 (11.8)	>60	692 (35.8)
Postgraduate	776 (40.1)	Outpatient working hours per week	
Undergraduate	857 (44.3)	≤ 8	584 (30.2)
Junior college	59 (3.1)	8–16	440 (22.8)
Other	14 (0.7)	16–24	440 (22.8)
Average monthly income (RMB)		24–40	268 (13.9)
≤ 5,000	376 (19.4)	>40	202 (10.4)
5,001–10,000	903 (46.7)	Number of outpatients serviced per day	
10,001–15,000	406 (21.0)	≤ 25	497 (25.7)
>15,000	249 (12.9)	26–40	582 (30.1)
Professional title		41–50	381 (19.7)
Senior	212 (11.0)	>50	474 (24.5)
Deputy senior	548 (28.3)	Amount of time spent per patient (minutes)	
Intermediate	699 (36.1)	≤ 5	601 (31.1)
Junior	450 (23.3)	5–10	867 (44.8)
Other	25 (1.3)	10–15	274 (14.2)
Working years in the current medical institution		>15	192 (9.9)
1–5	596 (30.8)	Self-rated health status	
6–10	503 (26.0)	Very poor	23 (1.2)
11–15	335 (17.3)	Poor	105 (5.4)
16–20	206 (10.7)	Fair	902 (46.6)
>20	294 (15.2)	Good	624 (32.3)
Area		Very good	280 (14.5)
Eastern China	735 (38.0)	Self-rated outpatient satisfaction	
Central China	685 (35.4)	Low	24 (1.2)
Western China	514 (26.6)	Medium	210 (10.9)
		High	1,700 (87.9)

**Table 2:** Mean mental workload scores (*n* = 1,934).

**Dimensions**	**Minimum**	**Maximum**	**Mean ± SD**	**Dimensions**	**Minimum**	**Maximum**	**Mean ± SD**
Mental demands	6.67	100.00	80.58 ± 15.17	Mental demands^*^	0.00	33.33	18.16 ± 8.67
Physical demands	5.00	100.00	72.28 ± 19.27	Physical demands^*^	0.00	33.33	8.36 ± 7.57
Temporal demands	0.00	100.00	72.81 ± 18.39	Temporal demands^*^	0.00	33.33	12.95 ± 7.15
Perceived risk	0.00	100.00	74.20 ± 20.15	Perceived risk^*^	0.00	33.33	15.51 ± 7.51
Frustration level	0.00	100.00	60.83 ± 23.14	Frustration level^*^	0.00	33.33	9.48 ± 7.83
Performance	0.00	100.00	27.79 ± 17.19	Performance^*^	0.00	33.33	3.55 ± 4.43
Total score^*^	17.11	100.00	68.01 ± 14.25				

* *Weighted score*.

### Identification of Different Subtypes of Physicians

According to model indexes, the best fitting LPA was the three-class model (Table 3), which had lower AIC (97069.375), BIC (97214.126) and ABIC (97131.523). The *p*-values of the LMR test (0.0017) and BLRT (<0.001) indicate this model was statistically significant at the α = 0.05; and a higher Entropy value (0.796) and proportion of physicians in the least class (16.4% > 10%) indicate the model was reliable and valid. In the three-class model, the average profile probabilities of physicians in each category ascribed to corresponding potential category ranged from 0.901 to 0.908, which further supports that the results of the three-class model were reliable and valid.

**Table 3:** Latent profile analysis models and fit indices.

**Model**	**AIC**	**BIC**	**ABIC**	**Entropy**	**LMR, *p*-value**	**BLRP, *p*-value**	**Proportion of physicians in the least class**
1	100945.121	101011.929	100973.805	–	–	–	–
2	98017.551	98123.331	98062.967	0.801	<0.001	<0.001	44.1%
3	97069.375	97214.126	97131.523	0.796	0.0017	<0.001	16.4%
4	96704.665	96888.388	96783.546	0.791	0.0089	<0.001	5.7%
5	96420.200	96642.894	96515.813	0.785	0.0390	<0.001	7.3%
6	96218.657	96480.323	96331.003	0.799	0.0251	<0.001	3.4%
7	96033.153	96333.790	96162.231	0.788	0.1847	<0.001	3.3%

*AIC, Akaike Information Criterion; BIC, Bayesian Information Criterion; ABIC, Sample-size-adjusted Bayesian Information Criterion; LMR, Lo-Mendell-Rubin Adjusted Likelihood Ratio Test; BLRT, Bootstrap Likelihood Ratio Test*.

**Figure 1:**
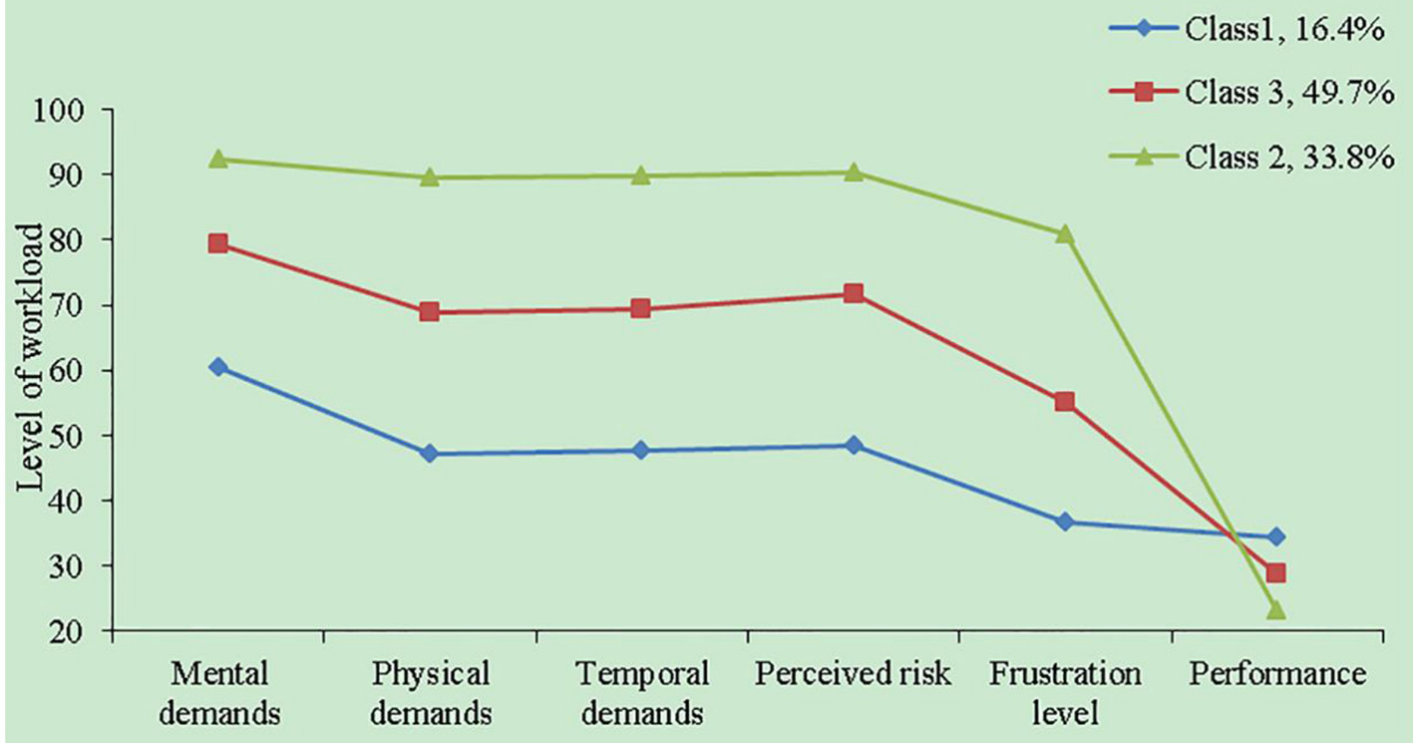
The latent profiles of the dimensions of the Chinese version of physician mental workload scale.

Figure 1 shows the subtypes of physicians (Classes 1, 2, and 3), their proportion (16.4, 33.8, 49.7%, respectively), and the mean levels of the mental workload and its dimensions, which can be distinguished as having relatively low (Class 1), medium (Class 3) and high (Class 2) levels of mental workload. The diagrams for Classes 3 and 1 shared similar patterns for the six dimensions of the physician mental workload scale. The scores for the dimensions of performance and frustration level were much lower than the other dimension scores in each subgroup. Specifically, Class 2 presented the highest task load and highest self-assessment of performance, named the “high workload and high self-assessment” subtype. Class 3 demonstrated medium levels of the six workload dimensions, named the “medium workload and medium self-assessment” subtype. However, Class 1 had the lowest scores in task load and the lowest self-assessment level of satisfaction with performance, named the “low workload and low self-assessment” subtype.

### Characteristics of Different Subtypes of Physicians

Chi-square tests (Supplementary Table S1) showed that there was a significant difference in the three subtypes for gender (χ^2^ = 15.925, *p* < 0.001), marital status (χ^2^ =12.726, *p* = 0.013), educational level (χ^2^ = 38.810, *p* < 0.001), average monthly income (χ^2^ = 16.635, *p* = 0.011), professional title (χ^2^ = 18.501, *p* = 0.018), hospital level (χ^2^ = 21.519, *p* = 0.001), personnel (χ^2^ = 16.768, *p* = 0.010), working hours per week (χ^2^ = 54.940, *p* < 0.001), outpatient working hours per week (χ^2^ = 24.806, *p* = 0.002), number of outpatients serviced per day (χ^2^ = 17.179, *p* = 0.009), amount of time spent per patient (χ^2^ = 17.714, *p* = 0.007), self-rated health status (χ^2^ = 60.977, *p* < 0.001), and self-rated outpatient satisfaction (χ^2^ = 45.659, *p* < 0.001). However, there was no significant difference in the three subtypes for area (χ^2^ = 0.722, *p* = 0.949) and department (χ^2^ = 13.024, *p* = 0.111).

When compared with those in the other subtypes, physicians in “high workload and high self-assessment” subtype tended to be those who were female, married, and personnel agency, those who had a postgraduate or undergraduate degrees, lower average monthly incomes, intermediate or deputy senior professional titles, worse self-rated health status, and better self-rated outpatient satisfaction, and those who worked in tertiary hospitals, worked more hours per week, saw more outpatients per day, spent more time on per patient and worked more hours per week in outpatient clinic.

Table 4 also presents the significant differences in mental workload and its dimensions among the three subtypes. The “high workload and high self-assessment” subtype was characterized by the highest scores on the dimensions of mental demands, physical demands, temporal demands, perceived risk, and frustration level, and the most successful performance of the task and the highest level of satisfaction with his/her performance. The “low workload and low self-assessment” subtype further distinguished itself from the “medium workload and medium self-assessment” subtype through lower scores on the five dimensions except for the performance dimension. Also, the “high workload and high self-assessment” subtype had the highest mean mental workload, followed by the “medium workload and medium self-assessment” subtype, and “low workload and low self-assessment” subtype, which can also be named “high mental workload” group, “medium mental workload” group and “low mental workload” group in turn.

**Table 4:** Comparisons of the different subtypes by mental workload.

**Dimensions**	**Class 1** **(*N* = 318) (Mean ± SD)**	**Class 3** **(*N* = 962) (Mean ± SD)**	**Class 2** **(*N* = 654) (Mean ± SD)**	***P*** **^a^-value**			
				**Overall**	**Class 1 vs. Class 3**	**Class 1 vs. Class 2**	**Class 3 vs. Class 2**
Mental demands	60.45 ± 15.77	79.24 ± 10.49	92.32 ± 7.77	<0.001	<0.001	<0.001	<0.001
Physical demands	47.26 ± 15.09	68.79 ± 13.31	89.57 ± 10.3	<0.001	<0.001	<0.001	<0.001
Temporal demands	47.77 ± 14.58	69.45 ± 11.69	89.92 ± 9.04	<0.001	<0.001	<0.001	<0.001
Perceived risk	48.43 ± 18.3	71.72 ± 14.8	90.38 ± 11.04	<0.001	<0.001	<0.001	<0.001
Frustration level	36.79 ± 16.44	55.19 ± 17.65	80.82 ± 16.36	<0.001	<0.001	<0.001	<0.001
Performance	34.32 ± 17.60	28.72 ± 14.99	23.24 ± 18.74	<0.001	<0.001	<0.001	<0.001
Total score	48.55 ± 8.20	65.96 ± 8.62	80.49 ± 10.62	<0.001	<0.001	<0.001	<0.001

*Class 1, “low workload and low self-assessment” subtype; Class 3, “medium workload and medium self-assessment” subtype; Class 2, “high workload and high self-assessment” subtype.*

a *ANOVA and post-hoc pairwise Bonferroni tests for the dimension indicators with a normal distribution*.

### Factors Associated With the Subtypes of Physicians

Multinomial logistic regression was used to identify the significant factors that influenced the subtypes of physicians in their mental workload. Using “high workload and high self-assessment” subtype (that is, “high mental workload” group) as the base outcome (reference), we had following results (Table 5).

**Table 5:** Multinomial logistic regression results: significant determinants of the subtypes of physicians in mental workload.

	**“Low workload and low self-assessment” subtype**			**“Medium workload and medium self-assessment” subtype**		
**Variables**	**β**	**Relative risk ratio (95% confidence interval)**	***p*** **-value**	**β**	**Relative risk ratio (95% confidence interval)**	***p*** **-value**
Gender (ref: Male)						
Female	−0.515	0.597 (0.424, 0.842)	0.003^***^	−0.261	0.770 (0.601, 0.987)	0.039^**^
Age (ref: 20–30years)						
31–40	0.824	2.279 (1.320, 3.935)	0.003^***^	0.302	1.352 (0.909, 2.011)	0.137
41–55	0.945	2.575 (1.290, 5.140)	0.007^***^	0.474	1.606 (0.967, 2.667)	0.067^*^
>55	0.326	1.385 (0.483, 3.976)	0.544	−0.354	0.702 (0.314, 1.569)	0.389
Marital status (ref: Unmarried)						
Married	−0.529	0.589 (0.351, 0.987)	0.044^*^	−0.277	0.758 (0.517, 1.111)	0.155
Divorced	−0.620	0.538 (0.170, 1.70)	0.290	−1.030	0.357 (0.147, 0.869)	0.023^**^
Widowed	N/A	N/A	N/A	N/A	N/A	N/A
Educational level (ref: PhD)						
Postgraduate	−0.630	0.533 (0.315, 0.900)	0.019^**^	−0.414	0.661 (0.442, 0.987)	0.043^**^
Undergraduate	−0.800	0.449 (0.250, 0.807)	0.007^***^	−0.186	0.831 (0.537, 1.285)	0.405
Junior college	2.554	1.291 (0.462, 3.610)	0.626	0.0972	1.102 (0.464, 2.615)	0.826
Other	N/A	N/A	N/A	N/A	N/A	N/A
Average monthly income (ref: 10,001–15,000 RBM)						
≤ 5,000	−0.239	0.787 (0.447, 1.387)	0.408	−0.252	0.777 (0.521, 1.160)	0.217
5,001–10,000	−0.324	0.723 (0.472, 1.107)	0.136	−0.381	0.682 (0.504, 0.924)	0.013^**^
>15,000	0.0499	1.051 (0.619, 1.785)	0.854	−0.0844	0.919 (0.617, 1.368)	0.678
Working years in the current medical institution (ref: 1–5 years)						
6–10	−0.454	0.635 (0.395, 1.020)	0.060^*^	−0.108	0.898 (0.635, 1.269)	0.542
11–15	−0.413	0.662 (0.380, 1.152)	0.145	−0.237	0.789 (0.524, 1.186)	0.254
16–20	−1.034	0.356 (0.179, 0.705)	0.003^***^	−0.659	0.518 (0.318, 0.843)	0.008^***^
>20	−0.563	0.569 (0.284, 1.143)	0.113	−0.327	0.721 (0.428, 1.216)	0.220
Hospital level (ref: Tertiary A hospital)						
Tertiary B hospital	0.262	1.300 (0.752, 2.246)	0.348	−0.240	0.787 (0.523, 1.185)	0.251
Secondary hospital	0.434	1.543 (1.007, 2.363)	0.046^**^	0.0822	1.086 (0.802, 1.470)	0.595
First-tier hospital	0.603	1.827 (0.593, 5.634)	0.294	−0.136	0.873 (0.336, 2.271)	0.781
Working hours per week (ref: ≤ 40)						
41–60	−0.884	0.413 (0.244, 0.699)	0.001^***^	−0.372	0.689 (0.440, 1.080)	0.104
>60	−1.599	0.202 (0.115, 0.356)	<0.001^***^	−0.682	0.505 (0.317, 0.805)	0.004^***^
Outpatient working hours per week (ref: ≤ 8)						
8–16	0.236	1.266 (0.829, 1.934)	0.275	0.364	1.439 (1.060, 1.955)	0.020^**^
16–24	0.269	1.309 (0.832, 2.058)	0.244	0.359	1.432 (1.036, 1.980)	0.029^**^
24–40	−0.0852	0.918 (0.540, 1.561)	0.753	0.0840	1.088 (0.741, 1.596)	0.668
>40	−0.598	0.550 (0.293, 1.035)	0.064^*^	0.0169	1.017 (0.682, 1.518)	0.934
Number of outpatients serviced per day (ref: ≤ 25)						
26–40	−0.848	0.428 (0.282, 0.651)	<0.001^***^	−0.341	0.711 (0.523, 0.966)	0.029^**^
41–50	−0.602	0.548 (0.343, 0.874)	0.0012^***^	−0.397	0.672 (0.476, 0.951)	0.025^**^
>50	−0.817	0.442 (0.271, 0.719)	0.001^***^	−0.413	0.661 (0.464, 0.941)	0.022^**^
Amount of time spent per patient (ref: 10–15min)						
≤ 5	0.383	1.467 (0.870, 2.474)	0.151	0.432	1.540 (1.051, 2.251)	0.026^**^
5–10	0.278	1.320 (0.817, 2.134)	0.256	0.431	1.538 (1.089, 2.171)	0.014^**^
>15	−0.0633	0.939 (0.499, 1.765)	0.844	0.364	1.439 (0.909, 2.278)	0.121
Self-rated health status (ref: Very good)						
Very poor	N/A	N/A	N/A	N/A	N/A	N/A
Poor	−1.377	0.252 (0.118, 0.541)	<0.001^***^	−0.642	0.526 (0.308, 0.899)	0.019^**^
Fair	−0.964	0.381 (0.247, 0.590)	<0.001^***^	−0.244	0.784 (0.556, 1.105)	0.164
Good	−0.163	0.850 (0.543, 1.329)	0.475	0.349	1.418 (0.986, 2.038)	0.060^*^
Self-rated outpatient satisfaction (ref: High)						
Low	2.376	10.758 (1.944, 59.521)	0.006^***^	1.266	3.547 (0.709, 17.471)	0.123
Fair	1.303	3.680 (2.320, 5.836)	<0.001^***^	0.548	1.730 (1.188, 2.518)	0.004^***^

*Base outcome: “high workload and high self-assessment” subtype;*

* *p < 0.1,*

** *p < 0.05,*

*** *p < 0.01*.

Female physicians were less likely to belong to “low workload and low self-assessment” [Relative Risk Ratio (RRR) = 0.597, *p* = 0.003] or “medium workload and medium self-assessment” (RRR = 0.770, *p* = 0.039) subtypes as compared with the odds of “high workload and high self-assessment” subtype. Physicians with higher age were more likely to belong to “low workload and low self-assessment” subtype; compared to those aged 20–30 years old, physicians aged 31–40 years old or 41–55 years old had a higher likelihood of belonging to “low workload and low self-assessment” subtype (RRR = 2.279, *p* = 0.003; RRR = 2.575, *p* = 0.007, respectively). Physicians being married were less likely than those being unmarried to belong to “low workload and low self-assessment” subtype (RRR = 0.589, *p* = 0.044). For educational level, physicians with lower education level were less likely to belong to “low workload and low self-assessment” subtype; compared to those with a PhD degree, physicians with postgraduate or undergraduate degrees had a lower likelihood of belonging to “low workload and low self-assessment” subtype (RRR = 0.533, *p* = 0.019; RRR = 0.449, *p* = 0.007, respectively), and physicians with a postgraduate degree also had a lower likelihood of belonging to “medium workload and medium self-assessment” subtype (RRR = 0.661, *p* = 0.043).

Physicians with an average monthly income of 5,001–10,000 RMB were less likely than those with an average monthly income of 10,001–15,000 RMB to belong to “medium workload and medium self-assessment” subtype (RRR = 0.682, *p* = 0.013) as compared with the odds of “high workload and high self-assessment” subtype. Compared to those who worked in the current medical institution for 1–5 years, physicians working 16–20 years in the current medical institution had a lower likelihood of belonging to “low workload and low self-assessment” (RRR = 0.356, *p* = 0.003) or “medium workload and medium self-assessment” (RRR = 0.518, *p* = 0.008) subtypes. Physicians in secondary hospitals were more likely than those in tertiary A hospitals to belong to “low workload and low self-assessment” subtype (RRR = 1.543, *p* = 0.046). For working hours per week, physicians who worked more hours per week were less likely to belong to “low workload and low self-assessment” or “medium workload and medium self-assessment” subtypes; compared to those with no more than 40 working hours per week, physicians with 41–60 or more than 60 working hours per week had a lower likelihood of belonging to “low workload and low self-assessment” subtype (RRR = 0.413, *p* = 0.001; RRR = 0.202, *p* < 0.001, respectively), and physicians who worked more than 60 h per week were less likely to belong to “medium workload and medium self-assessment” subtype (RRR = 0.505, *p* = 0.004).

For outpatient working hours per week, physicians who worked more than 40 h per week in outpatient clinics were less likely than those with no more than 8 outpatient working hours per week to belong to “low workload and low self-assessment” subtype (RRR = 0.505, *p* = 0.064 <0.10), whereas physicians who worked 8–16 or 16–24 h per week in outpatient clinics had a higher likelihood of belonging to “medium workload and medium self-assessment” subtype (RRR = 1.439, *p* = 0.020; RRR = 1.432, *p* = 0.029, respectively) as compared with the odds of “high workload and high self-assessment” subtype. For number of outpatients serviced per day, physicians with more outpatients serviced per day were less likely to belong to “low workload and low self-assessment” or “medium workload and medium self-assessment” subtypes; compared to physicians with no more than 25 outpatients serviced per day, physicians who saw more than 25 outpatients per day had a lower likelihood of belonging to “low workload and low self-assessment” (RRR = 0.428, *p* < 0.001; RRR = 0.548, *p* = 0.0012; RRR = 0.442, *p* = 0.001, respectively) or “medium workload and medium self-assessment” (RRR = 0.711, *p* = 0.029; RRR = 0.672, *p* = 0.025; RRR = 0.661, *p* = 0.022, respectively) subtypes. For amount of time spent per patient, physicians with less time spent per patient were more likely to belong to “medium workload and medium self-assessment” subtype; compared to physicians with 10–15 min spent per patient, physicians with no more than 5 or 5–10 min spent per patient had a higher likelihood of belonging to “medium workload and medium self-assessment” subtype (RRR = 1.540, *p* = 0.026; RRR = 1.538, *p* = 0.014, respectively).

For self-rated health status, physicians with worse self-rated health status were less likely to belong to “low workload and low self-assessment” subtype; compared to physicians who rated health status as “very good,” physicians who rated health status as “poor” or “fair” had a lower likelihood of belonging to “low workload and low self-assessment” subtype (RRR = 0.252, *p* < 0.001; RRR = 1.538, *p* < 0.001, respectively), and physicians who rated health status as “poor” were less likely to belong to “medium workload and medium self-assessment” subtype (RRR = 0.526, *p* = 0.019) as compared with the odds of “high workload and high self-assessment” subtype. However, physicians with lower self-rated outpatient satisfaction were more likely to belong to “low workload and low self-assessment” subtype; compared to physicians who rated outpatient satisfaction as “high,” physicians who rated outpatient satisfaction as “low” or “fair” had a higher likelihood of belonging to “low workload and low self-assessment” subtype (RRR = 10.758, *p* = 0.006; RRR = 3.680, *p* < 0.001, respectively), and physicians who rated outpatient satisfaction as “fair” had a higher likelihood of belonging to “medium workload and medium self-assessment” subtype (RRR = 1.730, *p* = 0.004). We also calculated the variance inflation factor to test the collinearity problem, and the variance inflation factor was <10 (1.07–2.65), indicating that there was no collinearity problem, and that the results of the model were reliable.

## Discussion

### Principal Findings

Overall, this survey study indicated a medium level of mental workload but with a relatively higher performance and self-rated outpatient satisfaction among Chinese physicians while performing communication work tasks characterized by direct patient interaction in outpatient clinics since the normalization of prevention and control of the COVID-19 pandemic in China compared to previous studies. About 33.8% of the participating physicians were identified as “high workload and high self-assessment” subtype, compared to 49.7% “medium workload and medium self-assessment” subtype and 16.4% “low workload and low self-assessment” subtype. The “high workload and high self-assessment” subtype with the highest level of mental workload, was characterized by the highest task load but the most successful performance of the task and the highest level of satisfaction with his/her performance. Previous studies often simply classify the mental workload groups using single indicators (overall mental workload) (25, 26).

Gender, age, marital status, educational level, average monthly income, working years in the current medical institution, hospital level, working hours per week, outpatient working hours per week, number of outpatients serviced per day, amount of time spent per patient, self-rated health status, and self-rated outpatient satisfaction were all significantly associated with the subtypes of mental workload among physicians while performing communication work tasks characterized by direct patient interaction in outpatient clinics.

### Comparison to Prior Studies

#### Mental Workload of Physicians

This survey study, to our knowledge, was an early study investigating the level of the mental workload of physicians and delineating the characteristics of the subtypes of these physicians in outpatient practice since the normalization of prevention and control of the COVID-19 pandemic in China. Current studies mainly addressed the assessments of mental workload of frontline nurses aiding in the COVID-19 epidemic; however, the mental workload level and its associated factors or characteristics among physicians likewise aiding in the COVID-19 pandemic, or working in outpatient settings after the pandemic were rarely reported (6, 9, 10). This study revealed that the total mean score of workload physicians perceived was 68.01 (SD = 14.25) while performing communication work tasks, which indicates a medium level of mental workload. The classes divided by the LPA showed that the total mean mental workload score in Class 2 (“high workload and high self-assessment” subtype, which accounted for 33.8% of the total sample) was 80.49 (SD = 10.62), which suggests a much higher level of mental workload than the mental workload reported not only in a study by Mazur et al. with radiation oncology professionals including physicians in America (range: 40–52) (26), and in a study conducted by Weigl et al. with hospital physicians in Germany (46.45 ± 17.29) (33) and in a study of Ariza et al. with general practitioners in England (28.7, 95% CI 23.3–34.0) (34) but also in a study by Ma et al. with physicians in outpatient departments in China (69.7 ± 11.5) (23) and in recent study conducted by Du et al. with frontline healthcare workers aiding in the COVID-19 pandemic in China (69.7 ± 16.4) (35). Regarding the objective workload, the latest data from National Health Commission of the People's Republic of China showed that the total number of annual outpatient visits in 2020 decreased 11.2% than that in 2019, whereas the medical service requirements of patients suppressed by the COVID-19 pandemic lasted for at least 5 months (18), indicating that a great number of accumulated medical demands may have been releasing in traditional hospitals after the rest of the time in 2020, and hence, the workloads physicians would undertake might be increased significantly over the same period since the normalization of prevention and control of the COVID-19 pandemic in China. Some physicians in outpatient practice might be exposed to the infection risks due to the sporadic outbreaks of COVID-19 in China, and might fear to infect their family, colleagues, and friends, and thus suffer from psychological pressure, anxiety and depression. Therefore, the challenges of the higher workloads and potential infection might seem sufficiently severe to increase the mental workload of physicians in outpatient practice.

As the graphs in the three-class model show, the scores for the dimensions of performance and frustration were much lower than the other dimension scores in each subgroup, whereas the dimensions of mental demands, perceived risk and temporal demands were main contributors of mental workload in this study, which contributed to the overall higher mental workload than that reported in previous studies (23, 26, 33–35). Another possible reason might be relevant to the fact that according to the definition of mental workload, mental workload could be determined by characteristics of the work task, the operator, and the environmental context or operational condition, where the work task was performed (11); and thus, communication work tasks characterized by direct patient interaction that mainly require physicians' brain resources, high-pressure workplace in outpatient clinics, and the participating physicians in this study mainly from high-level hospitals, where they tend to have heavy outpatient workloads (36), could together result in a higher level of mental workload reported in this study.

#### Determinants of Different Subtypes of Physicians

Findings of this study on determinants of the three subtypes of physicians showed that gender, age, marital status, educational level, average monthly income, working years in the current medical institution, hospital level, working hours per week, outpatient working hours per week, number of outpatients serviced per day, amount of time spent per patient, self-rated health status, and self-rated outpatient satisfaction were all the factors significantly associated with the subtypes of mental workload (that is, mental workload group) among physicians while performing communication work tasks characterized by direct patient interaction in outpatient clinics. A study by Du et al. also found that frontline health care workers aiding in the COVID-19 epidemic who perceived higher mental workload, tended to have higher education level and longer working years (35), and another study of Shan et al. revealed that frontline nurses with lower incomes were more likely to have a relatively low level of mental workload (8), and these two studies further suggested that there was no significant correlation between gender and mental workload, and similar conclusions were reported in several studies (37, 38). However, this study found that female physicians were more likely than male physicians to have a higher likelihood of belonging to those with high mental workload (that is, “high workload and high self-assessment” subtype) in outpatient practice; one possible reason for this difference might be relevant to the fact that compared to males, females among the participating physicians in this study might have longer outpatient working hours, when such a workload was combined with housework, children, and elderly care (39), which is supported by the finding from this study that physicians being married were more likely to have a high level of mental workload; another possible reason might be that female physicians showed more patience than male physicians in communication with their patients in clinics (40), thereby resulting in more consumption of their brain resources.

This study indicated that younger physicians (aged 20–30 years old) were more likely than other older groups (aged 31–55 years old) to have a relatively high level of mental workload while performing communication work tasks in outpatient practice; one possible explanation was that on the one hand, younger physicians, due to relative lack of experience, tended to have increased perceived workload (41), and on the other hand, these younger physicians in China were often likely to have lower professional titles, and thus to provide general outpatient services with a greater number of patients and have longer working hours, and moreover, the work pressure of scientific research owing to professional title assessment might also contribute to their workload to some extent (42).

Our study also found that physicians who saw more outpatients per day tended to have a relatively high level of mental workload while perform communication work tasks in outpatient clinics. Similar conclusions were reported in primary healthcare physicians by Orozco and Garcia (43) and in emergency department physicians by Prints et al. (44). Moreover, physicians with more time spent per patient in outpatient clinics were more likely to have a high level of mental workload, which is supported by the finding from Khori et al. that a longer mean consultation time of general physicians in Iran was significantly associated with their higher workload (45), however, another study conducted by Petek et al. found that physicians with absence of high workload in general practice in Slovenia tended to have longer consultation time (46); one possible explanation was that these physicians with more time spent per patient in this study might have higher professional titles, and mainly provide expert outpatient services for patients with intractable diseases, in which a greater number of their own brain resources were demanded while performing communication work tasks characterized by direct patient interaction in outpatient clinics. Compared to physicians in secondary hospitals, physicians in high-level hospitals (tertiary A hospitals) were more likely to have a higher likelihood of belonging to those with a high level of mental workload, because physicians in high-level hospitals on the one hand, tended to undertake a greater number of outpatient services, and on the other hand, provided higher quality medical services for outpatients (47), which means that a greater number of brain resources were demanded.

Health condition of physicians is a heated social problem in China. Previous studies have repeatedly emphasized the highly significant correlation between physicians' workload and their health (1, 2), and excessive mental workload can lead to not only serious health problems [for example, lower sleep quality (48), cardiovascular diseases and so on] for physicians (2) but also an inferior quality of care service (5) and further medical errors (49), thereby threatening patient safety. Our findings also showed that physicians with worse health status were more likely to have a relatively high level of perceived workload while performing communication work tasks characterized by direct patient interaction in outpatient clinics, indicating a major concern that should be focused on in this study. However, it was reported that there was a trend of dramatically increased workload for Chinese physicians from 1998–2016, potentially threatening their health and the quality of patient services (21). The analysis indicated that physicians with more working hours per week, and more working hours per week in outpatient clinics tended to have a high level of mental workload. Surprisingly, physicians had a higher level of mental workload but with better self-assessed performance in this study. These findings remind that hospital managers should further pay more attention to the effects of physicians' workload on their health to, in turn, prevent and reduce the risks of negative health outcomes, burnout and fatigue among physicians, and thereby improve the quality of medical services and patient safety in outpatient practice.

#### Characteristics of Different Subtypes of Physicians

Our findings also indicated the characteristics of the different subtypes of physicians in their mental workload, which could provide an opportunity for hospital mangers to develop targeted interventions for individual differences across physicians to prevent negative physical and psychological outcomes of physicians and improve their performance in outpatient clinics. Among the classes, Class 2 was referred to as the “high workload and high self-assessment” subtype, as these individuals tended to be female, married, younger, worse health status, have lower educational level and an average monthly income of 5,001–10,000 RMB, work in high-level hospitals and 16–20 years in the current medical institution, work more hours per week, work more hours in outpatient clinics, see more outpatients per day and spend more time per patient but with higher outpatient satisfaction. These characteristics represent that these individuals who had greater objective workloads in outpatient clinics were more likely to perceive a high level of mental workload, but with worse health condition. Meanwhile, their frustration level was high (80.82 ± 16.36). It can be speculated that the physical and psychological stress owing to heavier objective workload experienced by these physicians, and the nature of communication work tasks characterized by direct patient interaction that mainly require physicians' brain resources in outpatient practice may also result in job burnout. However, the mean self-reported outpatient work performance score was the lowest for this class, indicating that these physicians were the most successful in their performance or the most satisfied with their performance. Research indicated that there was a decreased consultation time (46) and self-rated performance (50), and an increased rate of severity grade of medical errors with increasing workload of physicians (51). These findings suggest that hospital managers should consider paying attention to physicians in “high workload and high self-assessment” subtype, monitor their workloads in real time and take measures to strengthen the management of their workload to, in turn, prevent and reduce negative physical and psychological outcomes of physicians and maintain their high work performance in outpatient practice. In addition, a study regarding the comparing the psychological impact of the COVID-19 outbreak between frontline and non-frontline medical workers in China reported that compared to non-frontline medical workers, frontline medical workers were more likely to suffer from mental health problems (i.e., anxiety, insomnia, and depressive symptoms) (9), and another study concerning factors associated with mental health outcomes among frontline and non-frontline healthcare workers in Oman during COVID-19 found that frontline healthcare workers were more likely than non-healthcare workers to report anxiety, stress and insomnia (52), and similar conclusions were reported in a narrative review regarding COVID-19-related mental health effects in the workplace that mental health problems, such as anxiety, depression, post-traumatic stress disorder (PTSD), suicidal ideas, and sleep disorders were more likely to affect the healthcare workers, especially those on the frontline (53); and a survey regarding mental health in frontline medical workers during the 2019 Novel Coronavirus Disease epidemic in China reveled that compared to those in other regions, frontline medical workers in Hubei Province (the epidemic center of the COVID-19 outbreak in 2019 in China) reported a high rate of symptoms of depression, anxiety, and insomnia, respectively (54). Moreover, some factors related to the risk of contagion in the organizational workplace and the adoption of preventive procedures [such as the lack personal protective equipment (PPE), the conflict between safety procedures and the desire to provide support, increased and heavy workload with multitasking as well as longer working hours, negative emotion of patients, distance of families, and fears of infection for themselves and their families] can deeper affect the mental well-being of these frontline healthcare workers during the COVID-19 epidemic (14, 53); and in response, when selecting interventions aimed at supporting frontline health workers' mental health, organizational, social, personal, and psychological factors might all be important reported in a systematic review (55), and thus, multiple organizational and work-related interventions (such as improvement of workplace infrastructures, the adoption of correct and shared anti-contagion measures), psychological support interventions (such as counseling and psychology services) and multifaceted interventions were recommended to help mitigate this scenario (53, 55). Furthermore, a systematic review further revealed that young age, and female gender, and heavy workload were the factors increasing the risks of suffering from post-traumatic stress symptoms in healthcare workers dealing with the COVID-19 pandemic (56), whereas negative mental health outcomes were associated with diminished work performance (57), and therefore, hospital managers should also pay more attention to these physicians in “high workload and high self-assessment” subtype, who were younger, female, participated in aiding in the COVID-19 pandemic, and even experienced mental health problems during the COVID-19 pandemic.

As for Classes 1 and 3 of the LPA model, both classes showed a similar pattern for the six dimensions of the Chinese version of physician mental workload scale. Class 1 showed the highest score in the performance dimension and the lowest task load, i.e., the “low workload and low self-assessment” subtype, whereas Class 3 showed a medium level for all mental workload dimensions, i.e., the “medium workload and medium self-assessment” subtype. Compared to those in Class 2, physicians in the two classes had a relatively low level of mental workload, and mainly shared the characteristics of being male, unmarried, older age, better health status, having higher educational level, working in the current medical institution for 1–5 years, working fewer hours per week, working fewer hours per week in outpatient clinics, seeing fewer outpatients per day, but with lower outpatient satisfaction, which indicates that these individuals were more likely to have relatively low outpatient workloads with a lot of room for work performance improvement. According to the characteristics in Classes 1 and 3, hospital managers should consider on the one hand distributing and increasing workloads for these physicians (especially in Class 3), especially when healthcare workers were urgently demanded for participating in aiding in the COVID-19 pandemic, and on the other hand, taking incentive measures to motivate these physicians to improve their work performance and the quality of medical services in outpatient practice.

## Limitations

This study has several limitations. First, although stratified convenient sampling was mainly used to recruit participants, due to the impact of the COVID-19 pandemic, we only employed an online questionnaire platform to collect data, and lower responsiveness was received in some selected hospitals, which may have impacted the generalizability of our conclusions, and thus, we generated a unique two-dimensional code of the electronic questionnaire for each hospital, and the outpatient managers in each selected hospital were invited and volunteered to play the role of the project manager in their hospitals in this survey. Second, data collection was self-reported by participating physicians via the online survey, and as a result, there was no guarantee that the participating physicians filled out the questionnaire just after finishing the provision of the outpatient services in outpatient practice, which might have a recall bias. Third, it is impossible to compare the differences in mental workload and its subtypes between frontline and non-frontline physicians since the question about whether physicians have participated in aiding in the COVID-19 pandemic in China, was not set up in the questionnaire. Fourth, the factors considered to differentiate the three subgroups were mainly based on the demographic variables in the demographic questionnaire, and therefore, further research should consider including more related factors to precisely identify the subtypes of mental workload among physicians.

## Conclusion

In general, participating physicians in our survey reported high levels of task load but good self-assessed performance while performing communication work tasks characterized by direct patient interaction in outpatient clinics since the normalization of prevention and control of the COVID-19 pandemic in China. About 33.8% of the participating physicians were identified as “high workload and high self-assessment” subtype, compared to 49.7% “medium workload and medium self-assessment” subtype and 16.4% “low workload and low self-assessment” subtype. Great individual variation among distinctive subtypes of mental workload of physicians exists. These findings can help provide a solid foundation for developing targeted interventions for individual differences across physicians regarding their mental workload. Therefore, we suggest that hospital managers should pay more attention to those physicians with the characteristics of the “high workload and high self-assessment” subtype and strengthen the management of the workload of this subtype of physicians to, in turn, reduce the risks of their mental health problems and maintain their high work performance in outpatient clinics. For physicians in other subtypes, we also suggest that the hospital managers should consider distributing and increasing workloads for these physicians (especially in “low workload and low self-assessment” subtype), especially when healthcare workers were urgently demanded for participating in aiding in the COVID-19 pandemic.

## Data Availability Statement

The datasets used and/or analyzed during the current study are available from the corresponding author on a reasonable request. Requests to access the datasets should be directed to hyh288@hotmail.com.

## Ethics Statement

The study was approved by the Ethics Committee of Tongji Medical College of Huazhong University of Science and Technology (No. IORG0003571); written informed consent from the patients/participants or patients/participants legal guardian/next of kin was not required to participate in this study in accordance with the national legislation and the institutional requirements.

## Author Contributions

YH designed the study, obtained funding, participated in the collection, and performed revisions of the manuscript. DL contributed to the acquisition, analysis and interpretation of survey data, and drafted the manuscript. HC participated in the collection, contributed to the interpretation of the results, and performed revisions of the manuscript. XZ, XW, and JL took part in the investigation and were involved in data cleaning. ZZ and SL were involved in data cleaning and contributed to the interpretation of the results. All authors have read and approved the final version of the manuscript.

## Funding

This study was supported by the National Natural Science Foundation of China (grant number 71774062).

## Conflict of Interest

The authors declare that the research was conducted in the absence of any commercial or financial relationships that could be construed as a potential conflict of interest.

## Publisher's Note

All claims expressed in this article are solely those of the authors and do not necessarily represent those of their affiliated organizations, or those of the publisher, the editors and the reviewers. Any product that may be evaluated in this article, or claim that may be made by its manufacturer, is not guaranteed or endorsed by the publisher.
