# Supplementary material for: Identifying the Subtypes and Characteristics of Mental Workload Among Chinese Physicians in Outpatient Practice: A Latent Profile Analysis
Source: Front Public Health. 2021 Nov 24;9:779262. doi: 10.3389/fpubh.2021.779262 (PMC8653799; doi:10.3389/fpubh.2021.779262)
Supplement: Supplementary file 1 [file Table_1.DOCX]

**TABLE S1 |** Comparisons of the mental workload of the three subtypes by demographic variables n (%).

| **Characteristics** | **‘low workload & low self- assessment’ subtype (N=318)** | **‘medium workload & medium self- assessment’ subtype (N=962)** | **‘high workload & high self-assessment’ subtype (N=654)** | **χ^2^** | **p** |
| --- | --- | --- | --- | --- | --- |
| Gender |  |  |  | 15.925 | <0.001^***^ |
| Male | 192 (60.4) | 540 (56.1) | 315 (48.2) |  |  |
| Female | 126 (39.6) | 422 (43.9) | 339 (51.8) |  |  |
| Age (years) |  |  |  | 6.912 | 0.329 |
| 20-30 | 71 (22.3) | 203 (21.2) | 159 (24.3) |  |  |
| 31-40 | 136 (42.8) | 426 (44.3) | 290 (44.3) |  |  |
| 41-55 | 97 (30.5) | 308 (32.0) | 182 (27.8) |  |  |
| $>$55 | 14 (4.4) | 25 (2.6) | 23 (3.5) |  |  |
| Marital status |  |  |  | 12.726 | 0.013^**^ |
| Unmarried | 64 (20.1) | 152 (15.8) | 89 (13.6) |  |  |
| Married | 242 (76.1) | 795 (82.6) | 548 (83.8) |  |  |
| Divorced | 8 (2.5) | 11 (1.1) | 17 (2.6) |  |  |
| Widowed | 4 (1.3) | 4 (0.4) | — |  |  |
| Educational level |  |  |  | 38.810 | <0.001^***^ |
| PhD | 48 (15.1) | 123 (12.8) | 57 (8.7) |  |  |
| Postgraduate | 124 (39.0) | 361 (37.5) | 291 (44.5) |  |  |
| Undergraduate | 120 (37.7) | 447 (46.5) | 290 (44.3) |  |  |
| Junior college | 20 (6.3) | 26 (2.7) | 13 (2.0) |  |  |
| Other | 6 (1.9) | 5 (0.5) | 3 (0.5) |  |  |
| Average monthly income (RMB) |  |  |  | 16.635 | 0.011^**^ |
| $\leq$5000 | 66 (20.8) | 186 (19.3) | 124 (19.0) |  |  |
| 5001-10000 | 135 (42.5) | 425 (44.2) | 343 (52.4) |  |  |
| 10001-15000 | 68 (21.4) | 221 (23.0) | 117 (17.9) |  |  |
| $>$15000 | 49 (15.4) | 130 (13.5) | 70 (10.7) |  |  |
| Professional title |  |  |  | 18.501 | 0.018^**^ |
| Senior | 40 (12.6) | 114 (11.9) | 58 (8.9) |  |  |
| Deputy Senior | 89 (28.0) | 262 (27.2) | 197 (30.1) |  |  |
| Intermediate | 95 (29.9) | 356 (37.0) | 248 (37.9) |  |  |
| Junior | 85 (26.7) | 220 (22.9) | 145 (22.2) |  |  |
| Other | 9 (2.8) | 10 (1.0) | 6 (0.9) | 11.700 | 0.165 |
| Working years |  |  |  |  |  |
| 1-5 | 85 (26.7) | 203 (21.1) | 140 (21.4) |  |  |
| 6-10 | 64 (20.1) | 225 (23.4) | 167 (25.5) |  |  |
| 11-15 | 54 (17.0) | 187 (19.4) | 126 (19.3) |  |  |
| 16-20 | 34 (10.7) | 114 (11.9) | 87 (13.3) |  |  |
| $>$20 | 81 (25.5) | 233 (24.2) | 134 (20.5) |  |  |
| Work years in the current medical institution |  |  |  | 12.946 | 0.114 |
| 1-5 | 114 (35.8) | 298 (31.0) | 184 (28.1) |  |  |
| 6-10 | 72 (22.6) | 248 (25.8) | 183 (28.0) |  |  |
| 11-15 | 53 (16.7) | 168 (17.5) | 114 (1.4) |  |  |
| 16-20 | 28 (8.8) | 94 (9.8) | 84 (12.8) |  |  |
| $>$20 | 51 (16.0) | 154 (16.0) | 89 (13.6) |  |  |
| Area |  |  |  | 0.722 | 0.949 |
| Eastern China | 123 (38.7) | 370 (38.5) | 242 (37.0) |  |  |
| Central China | 108 (34.0) | 341 (35.4) | 236 (36.1) |  |  |
| Western China | 87 (27.4) | 251 (26.1) | 176 (26.9) |  |  |
| Hospital level |  |  |  | 21.519 | 0.001^***^ |
| Tertiary A hospital | 186 (58.5) | 633 (65.8) | 415 (63.5) |  |  |
| Tertiary B hospital | 42 (13.2) | 87 (9.0) | 86 (13.1) |  |  |
| Secondary hospital | 76 (23.9) | 227 (23.6) | 144 (22.0) |  |  |
| First-tier hospital | 14 (4.4) | 15 (1.6) | 9 (1.4) |  |  |
| Hospital nature |  |  |  | 5.737 | 0.057^*^ |
| Public general hospital | 280 (88.1) | 908 (94.4) | 624 (95.4) |  |  |
| Public specialized hospital | 24 (7.5) | 45 (4.7) | 29 (4.4) |  |  |
| Private general hospital | 6 (1.9) | 5 (0.5) | — |  |  |
| Private specialized hospital | 8 (2.5) | 4 (0.4) | 1 (0.2) |  |  |
| Personnel |  |  |  | 16.768 | 0.010^**^ |
| Authorized strength | 202 (63.5) | 662 (68.8) | 449 (68.7) |  |  |
| Personnel agency | 36 (11.3) | 81 (8.4) | 84 (12.8) |  |  |
| Contract | 73 (23.0) | 206 (21.4) | 117 (17.9) |  |  |
| Other | 7 (2.2) | 13 (1.4) | 4 (0.6) |  |  |
| Department |  |  |  | 13.024 | 0.111 |
| Internal | 102 (32.1) | 298 (31.0) | 185 (28.3) |  |  |
| Surgical | 82 (25.8) | 244 (25.4) | 155 (23.7) |  |  |
| Obstetrics and Gynecology | 29 (9.1) | 84 (8.7) | 79 (12.1) |  |  |
| Pediatrics | 16 (5.0) | 82 (8.5) | 65 (9.9) |  |  |
| Other | 89 (28.0) | 254 (26.4) | 180 (170) |  |  |
| Working hours per week |  |  |  | 54.940 | <0.001^***^ |
| $\leq40$ | 59 (18.6) | 84 (8.7) | 37 (5.7) |  |  |
| 41-60 | 176 (55.3) | 542 (56.3) | 344 (52.6) |  |  |
| $>$60 | 83 (26.1) | 336 (34.9) | 273 (41.7) |  |  |
| Outpatient working hours per week |  |  |  | 24.806 | 0.002^***^ |
| $\leq$8 | 104 (32.7) | 309 (32.1) | 171 (26.1) |  |  |
| 8-16 | 70 (22.0) | 232 (24.1) | 138 (21.1) |  |  |
| 16-24 | 74 (23.3) | 188 (19.5) | 178 (27.2) |  |  |
| 24-40 | 49 (15.4) | 133 (13.8) | 86 (13.1) |  |  |
| $>$40 | 21 (6.6) | 100 (10.4) | 81 (12.4) |  |  |
| Number of outpatients serviced per day |  |  |  | 17.179 | 0.009^***^ |
| $\leq$25 | 102 (32.1) | 257 (26.7) | 138 (21.1) |  |  |
| 26-40 | 85 (26.7) | 279 (29.0) | 218 (33.3) |  |  |
| 41-50 | 65 (20.4) | 184 (19.1) | 132 (20.2) |  |  |
| $>$50 | 66 (20.8) | 242 (25.2) | 166 (25.4) |  |  |
| Amount of time spent per patient (minutes) |  |  |  |  |  |
| $\leq$5 | 103 (32.4) | 304 (31.6) | 194 (29.7) | 17.714 | 0.007^***^ |
| 5-10 | 138 (43.4) | 448 (46.6) | 281 (43.0) |  |  |
| 10-15 | 45 (14.2) | 108 (11.2) | 121 (18.5) |  |  |
| $>$15 | 32 (10.1) | 102 (10.6) | 58 (8.9) |  |  |
| Self-rated health status |  |  |  | 60.977 | <0.001^***^ |
| Very poor | 4 (1.3) | 8 (0.8) | 11 (1.7) |  |  |
| Poor | 14 (4.4) | 44 (4.6) | 47 (7.2) |  |  |
| Fair | 113 (35.5) | 438 (45.5) | 351 (53.7) |  |  |
| Good | 119 (37.4) | 352 (36.6) | 153 (23.4) |  |  |
| Very good | 68 (21.4) | 120 (12.5) | 92 (14.1) |  |  |
| Self-rated outpatient satisfaction |  |  |  | 45.659 | <0.001^***^ |
| Low | 13 (4.1) | 9 (0.9) | 2 (0.3) |  |  |
| Medium | 56 (17.6) | 103 (10.7) | 51 (7.8) |  |  |
| High | 249 (78.3) | 850 (88.4) | 601 (91.9) |  |  |
| *p < 0.1; **p < 0.05;***p < 0.01. |  |  |  |  |  |
